# Factors Influencing the Prevalence of PTSD Tendencies on Social Media During Emergencies

**DOI:** 10.3390/bs16091702

**Published:** 2026-09-21

**Authors:** Jingfang Liu, Chengyang Fu, Dingyun Lou

**Affiliations:** School of Management, Shanghai University, Shanghai 200444, China; jingfangliu@shu.edu.cn (J.L.); fu_chengyang@shu.edu.cn (C.F.)

**Keywords:** emergency, social media, post-traumatic stress disorder, factor

## Abstract

Post-traumatic stress disorder (PTSD) is a psychological condition that may develop after exposure to severe traumatic events, particularly in unexpected situations characterized by high uncertainty and stress. As social media has become an integral part of daily life, it serves as a platform for sharing personal experiences, emotional expressions, and public responses during emergencies. Grounded in Protection Motivation Theory, this study explores the factors associated with PTSD tendencies expressed on social media during emergencies. Using large-scale user-generated data from social media, posts exhibiting PTSD tendencies were identified through a machine-learning classifier based on semantic embeddings, and the Latent Dirichlet Allocation topic model was applied to extract relevant themes. A multiple linear regression model with Newey–West standard errors was then constructed to examine the factors related to the prevalence of PTSD tendencies. The results indicate that epidemic containment, protective measures, and health concern are negatively associated with the prevalence of PTSD tendencies on social media, whereas expressive negativity is positively associated with trauma-related expression. Focusing on the context of COVID-19, this study highlights the factors associated with PTSD tendencies on social media and underscores the importance of well-organized epidemic prevention and control measures and balanced information dissemination in mitigating the psychological impact during emergencies.

## 1. Introduction

Post-traumatic stress disorder (PTSD) is a prevalent psychological disorder that develops after exposure to severe traumatic events, including but not limited to warfare, natural disasters, life-threatening health crises, and bereavement. In recent years, research on PTSD has received growing academic attention, particularly in the context of emergency events. Emergencies are typically marked by high uncertainty and pervasive stress, which substantially increase the public’s risk of exposure to traumatic situations and subsequent psychological distress, making PTSD a critical issue in the field of public mental health during crises.

With the rapid development of information technology, social media has become an integral part of people’s daily lives. During crisis events, the public widely shares personal experiences, emotional expressions, and event-related responses and interactions via social media platforms. As one of the most active social media platforms in China, Weibo has a large user base and rich user-generated data resources. In emergencies such as large-scale epidemics, users tend to conduct in-depth discussions and emotional exchanges on the platform, leaving massive digital traces of their psychological states. These publicly available social media data not only reflect individuals’ mental status to a certain extent, but also provide a novel, large-scale perspective for researchers to capture the dynamic changes of public mental health in real time.

Extant studies have explored the prevalence patterns and influencing factors of PTSD from multiple dimensions. In traditional epidemiological research, scholars have conducted targeted investigations on trauma-exposed groups in different emergency scenarios. Nieh et al. (2019) proposed a methodological framework to analyze PTSD among healthcare workers following the 2018 Hualien earthquake in Taiwan, and explored potential risk factors for the disorder. Lee et al. (2021) identified various contributing factors of PTSD symptoms, and suggested that relevant findings could support the identification of high-risk groups and the development of psychological interventions to alleviate PTSD symptoms in disaster-affected populations. Focusing on the COVID-19 pandemic, Yoon et al. (2023) adopted a cross-sectional study design and found that direct involvement in patient care, witnessing patient deaths, inadequate nurse staffing, inconvenient electronic health record procedures, and COVID-19-related isolation experience were all associated with PTSD symptoms among medical staff.

Meanwhile, a growing body of research has begun to leverage social media data to expand the research scope of PTSD. Ismail et al. (2019) analyzed Twitter posts reflecting users’ psychological states, and employed transfer learning technology to identify cancer survivors with PTSD on social media. Levaot et al. (2021) found that providing help on social media after disasters predicts post-traumatic growth, but does not predict post-traumatic stress symptoms. Buodo et al. (2023) proposed that social motivation for social media use moderates the relationship between post-traumatic symptoms during COVID-19 lockdowns and distress improvement after the lockdown. Bhuptani et al. (2023) screened online disclosures of sexual victimization through the #MeToo hashtag on social media, and examined the association between online social reactions to such disclosures and post-traumatic stress disorder. Recent evidence demonstrates a significant nexus between digital media exposure and adverse mental health outcomes among youth and student populations navigating high-stress environments (Hoque et al., 2026).

Nevertheless, current research still has notable gaps. On the one hand, most existing studies on PTSD in emergency contexts focus on specific groups such as medical workers and disaster survivors, and there is a lack of research on the general public’s PTSD expression and its influencing factors based on open social media data in the Chinese context. On the other hand, existing social media-related studies mostly focus on single dimensions such as PTSD user identification or the correlation between social media use and trauma outcomes; few studies have combined automatic text classification and in-depth thematic analysis to systematically explore the thematic characteristics and potential influencing factors of PTSD-related public expression under emergency events.

To ground the empirical model within well-documented psychological literature, this study draws on Protection Motivation Theory (PMT; Rogers, 1975, 1983), a well-established framework for understanding how individuals respond to health threats. PMT proposes that, when facing a threat such as an epidemic, individuals form two types of cognitive appraisal: threat appraisal—the perceived severity of, and susceptibility to, the threat, which generates fear—and coping appraisal—the perceived efficacy of protective measures and one’s own ability to enact them, which reduces fear. This framework allows us to explain not only that epidemic-related factors relate to psychological states, but also why they do so.

To address the above research gaps, this study takes Weibo as the research scenario and focuses on PTSD-related public expression during emergencies, with the aim of exploring the factors associated with the prevalence of PTSD tendencies on social media. The study is carried out through two interlinked components: first, machine learning techniques are used to construct a classification model to screen posts with PTSD tendencies from large-scale Weibo posts; second, the Latent Dirichlet Allocation (LDA) topic modeling technique is applied to conduct in-depth thematic analysis on these PTSD-tendency posts, so as to clarify their thematic structure and identify potential influencing factors of PTSD reflected on social media under emergencies. The findings of this study can provide empirical support and decision-making reference for public mental health monitoring and targeted psychological intervention during emergencies.

## 2. Materials and Methods

### 2.1. Data

We retrieved the data of all of the posts from 19 January 2020 to 31 December 2022 under the topic of “epidemic” in Weibo, with a total of 319,697 posts, including the posting time, the body of the tweets, and other metrics. Given that the raw post dataset obtained from Weibo is usually characterized by large data volume but variable quality, meticulous cleaning work is required to filter out the noise and improve the reliability of the data. Specifically, we (1) removed posts clearly unrelated to the epidemic; (2) removed posts with incomplete fields or formatting errors; and (3) filtered out posts with excessive slang, internet jargon, or non-standard language to improve analytical accuracy. After this cleaning, the Weibo post dataset contained 243,223 user post samples. Data collection complied with the Weibo platform’s Terms of Service, and all analyses relied solely on anonymized, publicly available posts without involving sensitive personal information.

In addition, according to the official epidemic data released by China, the Chinese epidemic data from 19 January 2020 to 2 December 2022 is obtained after collation, with a data volume of 1049 days, containing indicators such as time, new cases, and so on.

### 2.2. Identification of PTSD-Tendency Posts

#### 2.2.1. Training Data and Labels

The classifier was trained on an open counseling question-and-answer (QA) corpus of 18,025 consultation records. Each record was pre-labeled by the corpus provider as PTSD-related (n = 236; 1.31%) or not PTSD-related (n = 17,789), with the label assigned on the basis of the consultation content rather than automated text matching, thereby avoiding label leakage. These labels served as the ground truth for post-level PTSD-tendency classification.

#### 2.2.2. Text Representation: Two Alternative Pipelines

To identify posts expressing trauma-related distress, we compared two text-representation pipelines: (1) a conventional bag-of-words approach in which term frequency–inverse document frequency (TF–IDF) features (unigrams and bigrams; min_df = 2, max_df = 0.9) were extracted and reduced to 40 dimensions via truncated singular value decomposition (SVD); and (2) a semantic approach in which each text was encoded into a 512-dimensional vector using a pre-trained Chinese BERT sentence-embedding model (Bidirectional Encoder Representations from Transformers; BAAI/bge-small-zh-v1.5) (Reimers & Gurevych, 2019), which captures semantic rather than surface lexical information (Devlin et al., 2019).

#### 2.2.3. Class-Imbalance Handling and Cross-Validation

Because PTSD-related records constitute only 1.31% of the corpus, we applied the Synthetic Minority Over-sampling Technique (SMOTE) to balance the training data (Chawla et al., 2002). Critically, SMOTE was applied within each cross-validation fold on the training split only, never on the full dataset before splitting (Kohavi, 1995); the held-out test fold was always kept in its original imbalanced distribution. This prevents synthetic samples from leaking into the test set. Model performance was evaluated with 5-fold stratified cross-validation, using the area under the ROC curve (AUC) as the primary, threshold-independent metric (Fawcett, 2006).

#### 2.2.4. Model Comparison and Selection

Four classifiers—logistic regression (LR), Gaussian naïve Bayes (NB), linear support vector machine (SVM), and random forest (RF)—were compared under both representation pipelines (Table 1). Under TF–IDF + SVD, all four classifiers performed near chance (AUC ≈ 0.49–0.53), indicating that surface lexical features carry little discriminative information for this task. Under BERT embeddings, all four improved substantially, with logistic regression achieving the highest cross-validated AUC (0.926 ± 0.017). Logistic regression was therefore selected as the final classifier owing to its superior, stable, and interpretable performance in this low-sample-size, high-dimensional setting. For completeness, we also report the full evaluation metrics for the selected BERT + logistic-regression model on a held-out 20% test set: binary precision = 0.100, recall = 0.617, F1 = 0.172; weighted precision = 0.983, recall = 0.923, F1 = 0.949; and AUC = 0.904. Because PTSD-related posts constitute only 1.31% of the corpus, binary precision is low at the default 0.5 threshold (reflecting a large number of false positives), whereas the weighted metrics are inflated by the dominant negative class. The threshold-independent AUC (0.926 in cross-validation) therefore best reflects the model’s discriminative ability, which is why we adopt a probability-weighted approach rather than a fixed threshold.

#### 2.2.5. Cross-Domain Validation on Weibo

Because the classifier was trained on a counseling corpus and applied to informal Weibo posts—a substantial domain shift—we manually validated its transferability. Two hundred Weibo posts were randomly sampled from three probability bands and independently judged by human coders as to whether they expressed trauma-related distress. The model excluded negative samples with 100% accuracy (0/50 low-probability posts were trauma-related), and 22% of the 100 high-probability posts (probability > 0.1) were judged as trauma-related—substantially above the random baseline. These results indicate that the model learned transferable semantic signals of trauma expression. Given the extreme class imbalance and domain shift, we adopted a probability-weighted approach (Section 2.5) rather than a fixed threshold: each post contributes its predicted PTSD-tendency probability, and the daily dependent variable is the mean probability across posts on that day.

### 2.3. LDA Thematic Analysis

To identify the thematic structure of trauma-related expression, we applied the Latent Dirichlet Allocation (LDA) topic-modeling technique to posts carrying elevated PTSD-tendency probabilities (Blei et al., 2003). Constructing the LDA model with a topic count of 4—chosen by comparing model perplexity across candidate topic numbers and considering the semantic interpretability of the resulting topics—we identified four topics and, based on the keywords generated by the model, inferred four factors that may be associated with PTSD tendencies expressed on social media during emergencies. The specific factors, their meanings, and representative keywords are shown in Table 2.

### 2.4. Research Hypothesis

We ground our hypotheses in PMT, which explains how individuals respond to health threats (Floyd et al., 2000). According to PMT, when facing a threat such as an epidemic, individuals form two types of cognitive appraisal: threat appraisal—the perceived severity of, and susceptibility to, the threat, which generates fear—and coping appraisal—the perceived efficacy of protective measures and one’s own ability to enact them, which reduces fear. Of the four LDA-derived themes (Table 2), we focus on the three that correspond most directly to coping appraisal—epidemic containment, protective measures, and health concern—together with expressive negativity, which reflects threat appraisal. Applied at the aggregate level, higher daily keyword densities of the three coping-appraisal factors reflect more thorough and well-organized prevention and health-monitoring behavior, whereas higher expressive negativity reflects higher threat appraisal. In addition, we controlled for three epidemic-related variables—cumulative confirmed cases, imported new cases, and cumulative cured cases—to account for the overall scale and trajectory of the epidemic. The details of the influencing factors are shown in Table 3.

#### 2.4.1. Impact of Epidemic Containment on the Prevalence of PTSD Tendencies

Epidemic containment captures the extent of epidemic prevention and control measures, such as home confinement, lockdown, and community control. From a PMT perspective, stringent and well-organized containment measures raise coping appraisal: they reduce virus transmission and the associated fear of infection and death, and provide the public with a sense of security and control (Rogers, 1983). When containment is more thorough—as reflected in more frequent discussion of confinement and lockdown—trauma-related distress should therefore be alleviated rather than aggravated. Accordingly, we propose the following:

**H1.**  *Epidemic containment is negatively associated with the prevalence of PTSD tendencies.*

#### 2.4.2. Impact of Protective Measures on the Prevalence of PTSD Tendencies

Protective measures capture preventive and protective behaviors such as nucleic acid testing, mask wearing, and disinfection. Under PMT, the widespread implementation of screening and protective measures enhances response efficacy—a core component of coping appraisal—which fosters a sense of being safeguarded and reduces fear and trauma-related distress (Rogers, 1975). We therefore expect higher protective-measure keyword density to be associated with lower trauma-related expression. Accordingly, we propose the following:

**H2.**  *Protective measures are negatively associated with the prevalence of PTSD tendencies.*

#### 2.4.3. Impact of Health Concern on the Prevalence of PTSD Tendencies

Health concern captures the public’s attention to health-related signals such as fever, body temperature, and physical health. Active attention to one’s health represents a form of self-efficacy and proactive coping; under PMT, higher self-efficacy is associated with lower fear, because individuals who feel able to monitor and manage the threat are less overwhelmed by it (Rogers, 1983). We therefore expect higher health-concern keyword density to be associated with lower trauma-related expression. Accordingly, we propose the following:

**H3.**  *Health concern is negatively associated with the prevalence of PTSD tendencies.*

#### 2.4.4. Impact of Express Negativity on the Prevalence of PTSD Tendencies

Express negativity captures the degree of negative emotion conveyed in social media posts. Negative emotions such as fear, pain, and despair are intrinsic to threat appraisal and are core components of trauma-related distress (Rogers, 1975). Higher negative expression therefore co-occurs with higher trauma-related expression. Accordingly, we propose the following:

**H4.**  *Express negativity is positively associated with the prevalence of PTSD tendencies.*

### 2.5. Variable Design and Selection

In this study, the term “PTSD tendencies” refers to trauma-related distress and coping expressions detectable in social media posts, as identified by a machine-learning classifier, and does not denote a formal clinical diagnosis of PTSD based on DSM-5 or ICD-11 criteria. The main goal of the model is to examine the factors associated with the prevalence of PTSD tendencies on social media during emergencies. Rather than assigning each post to a discrete category, we adopted a probability-weighted approach: each post contributes its predicted PTSD-tendency probability, and the dependent variable is accordingly measured as the daily mean PTSD-tendency probability—the average of these probabilities across all posts published on a given day. This continuous measure reflects the daily intensity of trauma-related expression on social media while avoiding the arbitrary selection of a classification threshold.

In addition to this, the details of the design and selection of the influencing factors in the model are shown in Table 4.

## 3. Results

### 3.1. Regression Estimation and Multicollinearity Test

The unit of analysis was the day (N = 1049 daily observations). Before estimation, we examined the stationarity of the variables using the Augmented Dickey–Fuller (ADF) test. The four focal variables—epidemic containment, protective measures, health concern, and express negativity—were all stationary (ADF *p* < 0.001), indicating no unit-root problem in the focal regressors. We then estimated the model by linear regression with Newey–West heteroskedasticity and autocorrelation consistent (HAC) standard errors (maximum lag of five) to correct for both heteroskedasticity and serial correlation; the Breusch–Godfrey test indicated the presence of serial correlation (χ^2^ = 47.22, *p* < 0.001), confirming that HAC standard errors are appropriate. Variance inflation factors (VIFs) were all below 10 (mean VIF = 2.72), indicating no problematic multicollinearity. Table 5 reports the full estimation results, including coefficients, HAC standard errors, 95% confidence intervals, and significance levels.

### 3.2. Robustness Check

To mitigate the influence of extreme values on the statistical analysis, the dependent variable was Winsorized at the 1st and 99th percentiles, and the model was re-estimated with Newey–West standard errors. As reported in Table 6, all focal variables retained their signs and statistical significance, confirming the robustness of the findings.

Second, we addressed the concern that the text-derived predictors and the text-derived outcome share lexical structure, which could inflate the estimated associations, by breaking their shared-text dependency. Within each day, posts were randomly assigned to two disjoint halves, and this procedure was repeated across 1000 random partitions; the dependent variable was computed from one half and the keyword-density predictors from the other, while expressive negativity was computed from all posts. Table 7 summarizes the results across the 1000 partitions. The hypothesized direction was preserved in 96.7–100% of the partitions for all four focal variables, with no systematic sign reversal. Expressive negativity and health concern were significant in the large majority of partitions, whereas epidemic containment and protective measures were significant in a smaller share, suggesting that a portion of their primary-specification magnitude reflected shared lexical structure; these two associations are therefore interpreted as day-level co-occurrence, and their primary-specification magnitudes are treated with appropriate caution.

## 4. Discussion

Based on the model results, the details of the hypothesis validation are shown in Table 8.

The model results are directionally consistent with all four hypotheses, although the robustness of the associations differs across factors: health concern and expressive negativity remained robust in the shared-text partition test, whereas epidemic containment and protective measures were attenuated. Consistent with Protection Motivation Theory, epidemic containment was negatively associated with the prevalence of PTSD tendencies, suggesting that more thorough containment measures—such as home confinement, lockdown, and community control—alleviate rather than aggravate trauma-related distress. This finding aligns with the PMT prediction that stringent prevention measures raise coping appraisal by reducing virus transmission and the associated fear of infection and death.

Protective measures were also negatively associated with trauma-related expression. The widespread implementation of screening and protective behaviors, such as nucleic acid testing, mask wearing, and disinfection, appears to enhance response efficacy and foster a sense of being safeguarded, thereby reducing fear. Similarly, health concern was negatively associated with trauma-related expression, indicating that the public’s active attention to health signals—such as fever and body temperature—reflects proactive coping and self-efficacy rather than passive vulnerability.

By contrast, expressive negativity was positively associated with the prevalence of PTSD tendencies. This finding is consistent with the view that negative emotions such as fear, pain, and despair are intrinsic to threat appraisal and co-occur with trauma-related distress; higher negative expression therefore marks higher trauma-related expression rather than serving as a protective release.

It should be noted that the robustness of these associations differs across factors. In the shared-text partition test (Section 3.2), health concern and expressive negativity remained significant in the majority of partitions, whereas epidemic containment and protective measures were attenuated. The latter two associations should therefore be interpreted as day-level co-occurrence whose primary-specification magnitudes may partly reflect shared lexical structure rather than robust determinants.

Online platforms also played a dual role during the epidemic, consistent with the two appraisal processes in Protection Motivation Theory. On the one hand, information overload and repeated exposure to negative news can heighten threat appraisal, increasing anxiety and trauma-related distress (Thompson et al., 2019; Garfin et al., 2020). On the other hand, active emotional expression and engagement with online peer communities can strengthen coping appraisal, serving as channels for emotional release and social support. Although expressive negativity was positively associated with trauma-related expression, the supportive and cathartic functions of online communities may nonetheless help individuals cope with distress. Future research could disentangle these two roles.

Several limitations should be noted. First, because the data were derived from public Weibo posts, we could not observe proximal social environments such as family dynamics, household support structures, or family functioning; these factors may buffer or exacerbate psychological distress (Leal Maridueña et al., 2025) and warrant investigation in future work. Second, our measures are aggregate-level proxies: the keyword-density variables capture the prevalence of epidemic-related topics rather than individuals’ actual cognitive appraisals, and the sentiment-based measure captures expressed emotion rather than internal states. Third, the analysis relies on self-reported, cross-sectional social-media expressions rather than clinical assessments, so the findings capture online trauma-related expression rather than diagnosed PTSD. Fourth, the classifier was trained on a counseling corpus, and its transfer to Weibo—though manually validated—may still introduce measurement error.

## 5. Conclusions

This study provides new insights into the factors associated with trauma-related expression on social media during emergencies. Grounded in Protection Motivation Theory, the findings reveal that more active health concern is associated with lower levels of trauma-related expression and higher expressive negativity is associated with higher trauma-related expression; epidemic containment and protective measures showed associations in the expected direction, but these were less robust once the shared-text dependency between predictors and outcome was removed. These results suggest that well-organized prevention and control measures—by reducing virus transmission and fostering a sense of security—may help alleviate public psychological distress during health emergencies, although the containment- and protective-measure associations should be interpreted with caution.

The findings also underscore the value of monitoring social-media expression as a real-time indicator of public mental health. Because expressive negativity co-occurs with trauma-related distress, it can serve as an early signal for identifying populations in need of psychological support. Overall, this study advances understanding of how epidemic prevention and control measures relate to public trauma-related expression, and highlights the importance of balanced information dissemination and targeted psychological support during large-scale health emergencies.

These findings have actionable implications for institutions, educators, and mental health professionals. First, because health concern—and, to a more qualified extent, epidemic containment and protective measures—were associated with lower trauma-related expression, public-health agencies should communicate prevention measures clearly and ensure they are perceived as effective, thereby strengthening the public’s coping appraisal and reducing fear. Second, institutions and platform managers can monitor expressive negativity on social media as a real-time signal of elevated trauma-related distress, enabling early and targeted psychological support during crises. Third, mental health professionals can leverage online peer communities to deliver targeted support, paying particular attention to users expressing high levels of negative emotion, as these expressions are associated with elevated trauma-related distress.

## Figures and Tables

**Table 1:** Comparison of classifiers under two text-representation pipelines (5-fold cross-validated AUC).

Classifier	TF–IDF + SVD	BERT
Logistic Regression	0.493 ± 0.050	0.926 ± 0.017
Naive Bayes	0.500 ± 0.038	0.869 ± 0.009
SVM (linear)	0.488 ± 0.029	0.909 ± 0.021
Random Forest	0.527 ± 0.034	0.914 ± 0.023

**Table 2:** Themes—impact factors.

Factor	Meaning	Keyword
Epidemic Containment	Restrictions and control measures imposed to contain the epidemic	Home, prevention, control, containment, isolation, restriction
Protective Measures	Preventive and protective behaviors such as testing, masking, and disinfection	Nucleic acid testing, mask, antigen, sterilization, sampling
Event Development Attention	Level of concern about the development of emergency	New cases, confirmed cases, asymptomatic, suspected cases, close contact
Health Concern	Attention to health-related signals such as fever and body temperature	Fever, health, cold, body temperature

**Table 3:** Details of influencing factors.

Factor	Meaning
Epidemic Containment	Restrictions and control measures imposed to contain the epidemic
Protective Measures	Preventive and protective behaviors such as testing, masking, and disinfection
Health Concern	Attention to health-related signals such as fever and body temperature
Express Negativity	The extent to which people express negative content through social media

**Table 4:** Variable design and selection details.

Variable	Measurement Method
Epidemic Containment	Normalized word density of epidemic-containment keywords (the daily count of these keywords divided by the daily total word count)
Protective Measures	Normalized word density of protective-measure keywords (daily keyword count/daily total word count)
Health Concern	Normalized word density of health-concern keywords (daily keyword count/daily total word count)
Express Negativity	Emotional polarity value computed using the HowNet Chinese sentiment lexicon (via the cnsenti package, v0.0.7), defined per post as (negative emotion words − positive emotion words)/total words, then averaged across posts within each day
Cumulative Confirmed Cases	Official state data on the cumulative number of confirmed cases
Imported New Cases	Official state data on the daily number of imported new cases
Cumulative Cured Cases	Official state data on the cumulative number of cured cases

**Table 5:** Regression results with Newey–West (HAC) standard errors with a maximum lag of five (N = 1049).

Variable	Coefficient	HAC SE	t	95% CI
Epidemic Containment	−0.0747 ***	0.0226	−3.31	[−0.119, −0.030]
Protective Measures	−0.0323 **	0.0155	−2.08	[−0.063, −0.002]
Health Concern	−0.0632 ***	0.0245	−2.58	[−0.111, −0.015]
Express Negativity	0.0568 ***	0.0186	3.06	[0.020, 0.093]
Cumulative Confirmed Cases	2.43 × 10^−10^ ***	6.81 × 10^−11^	3.57	[1.10, 3.77] × 10^−10^
Imported New Cases	−7.18 × 10^−6^ **	2.87 × 10^−6^	−2.50	[−1.28, −0.16] × 10^−5^
Cumulative Cured Cases	−3.93 × 10^−9^ **	1.77 × 10^−9^	−2.22	[−7.39, −0.46] × 10^−9^
_cons	0.0067 ***	0.0007	10.33	[0.0055, 0.0080]
R^2^ = 0.083; Adjusted R^2^ = 0.076; F(6, 1041) = 6.13, *p* < 0.001; Durbin–Watson = 1.673.				

Note. The keyword-based predictors (Epidemic Containment, Protective Measures, and Health Concern) are measured as normalized word densities (the daily count of theme keywords divided by the daily total word count); Expressive Negativity is computed using the HowNet Chinese sentiment lexicon (via the cnsenti package). Standard errors are Newey–West heteroskedasticity- and autocorrelation-consistent (HAC) with a maximum lag of five. ** *p* < 0.05, *** *p* < 0.01.

**Table 6:** Robustness check: regression results after Winsorization (Newey–West standard errors, N = 1049).

Variable	Coefficient	HAC SE	t
Epidemic Containment	−0.0610 ***	0.0177	−3.45
Protective Measures	−0.0294 **	0.0141	−2.08
Health Concern	−0.0574 **	0.0228	−2.51
Express Negativity	0.0527 ***	0.0162	3.25
Cumulative Confirmed Cases	2.47 × 10^−10^ ***	6.91 × 10^−11^	3.58
Imported New Cases	−7.07 × 10^−6^ **	2.86 × 10^−6^	−2.47
Cumulative Cured Cases	−3.67 × 10^−9^ **	1.80 × 10^−9^	−2.04
_cons	0.0063 ***	0.0005	12.59
F(6, 1041) = 7.54, *p* < 0.001.			

Note. The keyword-based predictors (Epidemic Containment, Protective Measures, and Health Concern) are measured as normalized word densities (the daily count of theme keywords divided by the daily total word count); Expressive Negativity is computed using the HowNet Chinese sentiment lexicon (via the cnsenti package). Standard errors are Newey–West heteroskedasticity- and autocorrelation-consistent (HAC) with a maximum lag of five. ** *p* < 0.05, *** *p* < 0.01.

**Table 7:** Shared-text partition robustness check across 1000 random partitions.

Variable	Hyp. Direction	Direction Preserved	Significant (*p* < 0.05)	Significant (*p* < 0.10)	Mean Coefficient
Epidemic Containment	Negative	98.4%	40.4%	56.8%	−0.044
Protective Measures	Negative	96.7%	30.9%	43.2%	−0.027
Health Concern	Negative	99.5%	60.1%	71.8%	−0.059
Express Negativity	Positive	100.0%	84.8%	95.0%	+0.054

Note. Within each day, posts were randomly split into two disjoint halves; the dependent variable was computed from one half and the three keyword-density predictors from the other, while expressive negativity was computed from all posts. Epidemic Containment, Protective Measures, and Health Concern are measured as normalized word densities (the daily count of theme keywords divided by the daily total word count), and Expressive Negativity is computed using the HowNet Chinese sentiment lexicon (via the cnsenti package). Coefficients are from OLS with Newey–West heteroskedasticity- and autocorrelation-consistent (HAC) standard errors with a maximum lag of five. “Significant” refers to the proportion of the 1000 partitions in which the coefficient reached the indicated significance level.

**Table 8:** Results of hypothesis validation.

Hypothesis	Meaning	Result
H1	Epidemic containment is negatively associated with the prevalence of PTSD tendencies	Partially supported
H2	Protective measures are negatively associated with the prevalence of PTSD tendencies	Partially supported
H3	Health concern is negatively associated with the prevalence of PTSD tendencies	Supported
H4	Express negativity is positively associated with the prevalence of PTSD tendencies	Supported

## Data Availability

The datasets generated and/or analyzed in the current study are available from the corresponding author on reasonable requests.

## Abbreviations

The following abbreviations are used in this manuscript:

PTSD	Post-traumatic stress disorder
PMT	Protection Motivation Theory
QA	Question-and-answer
TF–IDF	Term frequency–inverse document frequency
SVD	Singular value decomposition
BERT	Bidirectional Encoder Representations from Transformers
SMOTE	Synthetic Minority Over-sampling Technique
LDA	Latent dirichlet allocation
ADF	Augmented Dickey–Fuller
HAC	Heteroskedasticity and Autocorrelation Consistent
